# Open Surgical Simulation in the Minimally Invasive Era: A Binational Bowel Anastomosis Training Program

**DOI:** 10.7759/cureus.88110

**Published:** 2025-07-16

**Authors:** Franco Badaloni, Maria Jimena Alaniz Alday, Carolina Mendy, Santiago Pla, Juan Manuel Sanguinetti, Gabriel Eduardo Gondolesi, Pablo Barros-Schelotto, Andrés Fraile

**Affiliations:** 1 General Surgery, Favaloro Foundation University Hospital, Buenos Aires, ARG; 2 Surgery, Maldonado Medical Union FEMI, Medical College of Uruguay, Maldonado, URY

**Keywords:** open surgery, simulation based learning, skills and simulation training, small bowel anastomosis, surgical simulation

## Abstract

Introduction: Acquiring surgical skills is a mandatory factor in a surgeon's training, with simulation being a key tool to achieve this goal. Laparoscopic technical simulation is widely evaluated and validated, relegating open surgery; moreover, most surgical centers do not have a structured training program. The aim of this study was to evaluate a binational training program for performing bowel anastomosis in open surgery using a reproducible evaluation system.

Methods: We conducted a quasi-experimental study involving 20 participants who were divided into four groups according to their level of training. Supervised sessions using inanimate models were carried out. Surgical skills (Objective Structured Assessment of Technical Skill (OSATS)), operative time, hermeticity and permeability, and perception of autonomy were evaluated using an ex vivo bovine model. Statistical analysis was performed using Chi^2^, t-test, and Wilcoxon rank-sum test with SPSS version 25.0 (IBM Corp., Armonk, NY, USA); significance was set at α < 0.05.

Results: The average time to complete an anastomosis decreased from 34 to 20 minutes for the entire sample (p<0.001), with the greatest improvement observed between students and residents (p<0.001). Surgical skills measured by OSATS score increased across all participants from 23.10 to 28.75 (p<0.001), with significant gains in students (p=0.04) and residents (p=0.04). Permeability improved significantly in the full cohort (p<0.02), while hermeticity showed no overall statistical difference. Perceived autonomy increased among students and residents.

Conclusions: This study supports the implementation of structured, simulation-based training programs in open surgery as an effective strategy to improve technical skills and confidence during surgical education. Despite the small sample size, these findings highlight the value of reinforcing open surgical competencies in the minimally invasive era.

## Introduction

Acquiring surgical skills is a central and mandatory factor in a surgeon's formation. The evolution of operative techniques, the emergence of new technologies, the need for good outcomes, and patient safety have led to a notable reduction in training opportunities. It is currently unacceptable and inappropriate for surgeons at any level of expertise to practice new skills on patients, even with explicit consent [1,2].

In response to this problem, there is a trend toward using simulation for training surgical skills, with many successful experiences aimed at shortening the learning curve [3]. Simulation provides the trainee surgeon better overall performance, greater efficiency in time and movement, fewer errors, and better results in practice [4,5].

For simulation to be cost-effective and efficient, it must be framed within the concept of deliberate practice. It requires the guidance and interaction of an instructor capable of setting goals, breaking down skills into concepts, providing feedback, and offering the opportunity to learn from mistakes. In this way, the simulation laboratory provides a safe space where the surgeon can perform, repeat, and execute procedures, integrating them with reflective assistance [6-10].

The reduction in the volume of open surgeries performed by trainee surgeons, a direct consequence of the increase in laparoscopic surgery, is one of the main concerns for training systems. Open surgery is the foundation on which a surgeon's training is built, and the incorporation of new techniques and innovations does not change its basic importance [8].

Multiple simulation scenarios have been developed with specific goals. The creation of intestinal anastomoses is a complex procedure that requires delicate tissue handling and advanced suturing skills [11]. For this reason, we aimed to evaluate a bowel anastomosis training program in open surgery using a reproducible evaluation system through cooperation between two surgical centers from Uruguay and Argentina.

## Materials and methods

The study design is quasi-experimental. It was conducted at the Surgical Simulation Laboratory of the Favaloro Foundation University Hospital from April 2023 to March 2024. Favaloro Foundation University Hospital Ethics Committee issued approval CCE No. 015/CBE No. 217.

The study included 20 participants, evenly divided into four groups based on their training level and professional experience: (1) medical students with no prior surgical training; (2) general surgery residents; (3) early career general surgeons with less than five years of independent surgical practice (surgeons); and (4) advanced general surgeons with five or more years of experience, considered experts. Thus, four groups were formed: medical students (M), residents (R), surgeons (S), and experts (E). For part of the analysis, they were regrouped into two groups by experience: students and residents on one side and surgeons and experts on the other.

Training program

To identify and address the fundamental concepts and skills required to perform an intestinal anastomosis, the first step was to conduct interviews with highly experienced surgeons and trainee surgeons. With the resulting information, we designed a conceptually and progressively challenging training program divided into stages, with the final goal being the creation of a two-plane, side-to-side intestinal anastomosis in an ex vivo bovine model. The concepts covered were knots and sutures, thread adjustment and tying, needle handling, tissue handling, symmetry and alignment, economy of movement, point depth, and three-dimensionality, through simulated scenarios with inanimate models.

Each participant underwent eight training sessions over four weeks. Except for the first and last sessions, considered evaluation instances, each included introductory videos for each station, a briefing, 45 minutes of practice with an instructor, and a debriefing.

The model used was a 25-35cm segment of bovine small intestine. To increase fidelity, the scenario includes an aluminum base covered by a surgical drape, with a 25cm diameter Alexis® wound protector/retractor device and 9cm height supports providing surgical depth. The intestinal anastomosis was performed side-to-side and in two planes. Silk 2-0 sutures were used for the seromuscular plane, polypropylene 4-0 double-needle for the full-thickness plane, gloves, needle holder, scalpel, scissors, dissecting pick-ups, curved Crile forceps, and an LED light ring with a cellphone holder.

Evaluation

Surgical skills, operative time, perception of autonomy, and the hermeticity and permeability of the intestinal anastomosis were evaluated in two instances: one before training (pre-test) and another at the end (post-test).

To maintain anonymity, encrypted videos of each pre-test and post-test were recorded, positioning the camera so that only latex-gloved hands were visible, and without audio. Surgical skills and time were cross-evaluated by a trained instructor from Maldonado Medical Union, Uruguay using the validated Objective Structured Assessment of Technical Skill (OSATS) scale [12-14], which includes seven items: respect for tissue, time and motion, instrument handling, instrument knowledge, use of assistant, surgical flow, and advance planning, and procedure-specific knowledge.

Participants' perception of autonomy was evaluated using a seven-point Likert scale to answer the question, "How prepared do you feel to perform an anastomosis?" (Appendix 1).

The permeability and hermeticity of the intestinal anastomoses were evaluated by injecting low-pressure and burst pressure saline solution, respectively, by the Favaloro Foundation University Hospital instructor [11]. Permeability was considered the passage of saline solution through the anastomosis, and hermeticity was the absence of leaks after maximum pressure instillation with a Bonneau syringe, opposing the closure of the distal cavity with a Crile clamp.

Statistical analysis

Categorical variables were described with absolute and relative values, and quantitative variables with mean and standard deviation or median and interquartile range, according to distribution.

Non-categorical variables were compared using the chi-squared test. Normality was tested on quantitative variables using the Shapiro-Wilk test, then compared using the t-test for related samples across the four groups by experience, and independent samples to analyze the regrouped students/residents and surgeons/experts categories. The Wilcoxon rank test was used for the comparison of quantitative variables by training. Data were analyzed using SPSS version 25.0 (IBM Corp., Armonk, NY, USA), with statistical significance set at α < 0.05.

## Results

The evaluated variables for the entire sample and by training are described below (Table 1).

**Table 1:** Description of pre- and post-training variables. M: Medical Students; R: Residents; S: Surgeons (less than five years of experience); E: Experts (five or more years of experience). SD: Standard Deviation. IQR: Interquartile Range. * Mean (SD) for the entire sample / Median (IQR) when described by training. ** Absolute value is reported when described by training.

Variable	Pre-training					Post-training				
	Total	M	R	S	E	Total	M	R	S	E
Time (min) mean (SD)/Median (IQR)*	34 (16)	54 (12)	46 (5)	18 (5)	16 (4)	20 (10)	31 (9)	20 (4)	15 (2)	13 (1)
Hermeticity (Yes) n (%) **	16 (80)	2	4	5	5	20 (100)	5	5	5	5
Permeability (Yes) n (%) **	17 (85)	3	4	5	5	20 (100)	5	5	5	5
Skills mean (SD)/Median (IQR)*	23.10 (7.82)	13 (4)	18 (5)	29 (2)	32 (2)	28.75 (3.75)	24 (1)	26 (1)	31 (2)	33 (1)

**Figure 1:**
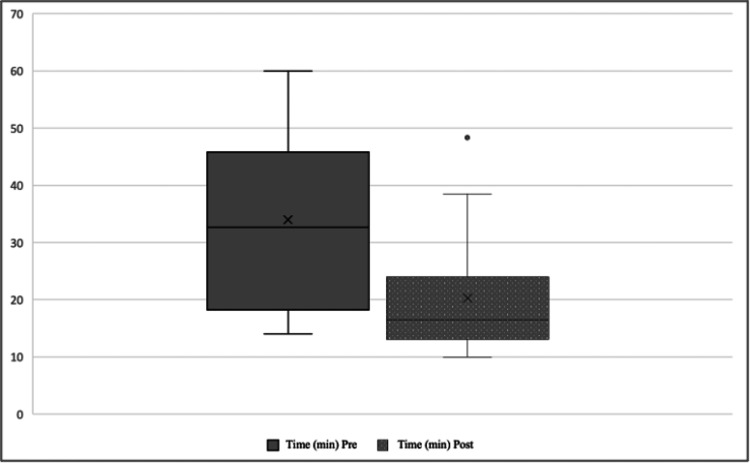
Mean time pre- and post-training.

The average time to perform an anastomosis for the entire sample decreased significantly from 34 minutes to 20 minutes (p<0.001) (Figure 1).

**Figure 2:**
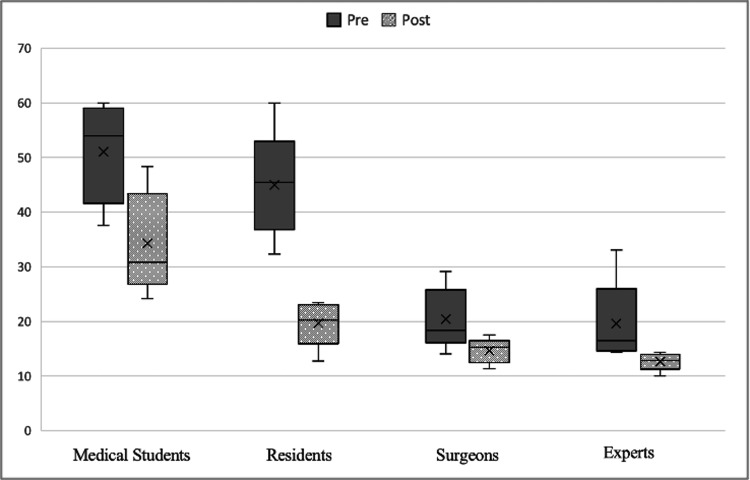
Mean time pre- and post-training according to level of experience.

When comparing the mean pre- and post-training time according to training, it was observed that it significantly decreased in all four groups (p<0.043) (Figure 2).

**Figure 3:**
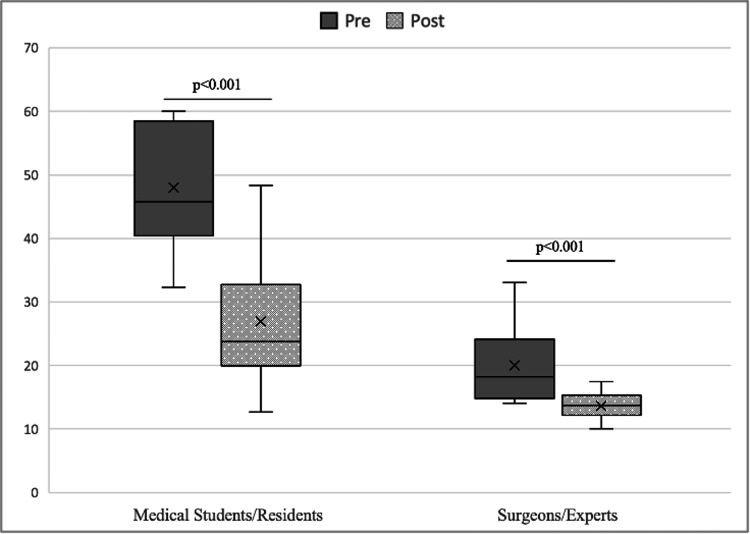
Average time pre- and post-training regrouped according to experience.

When regrouping the participants into the categories students/residents and surgeons/experts, the mean pre- and post-test time was significant between groups (p<0.001). The same occurred in the intra-group analysis, with greater significance in the case of students/residents (p<0.001) (Figure 3).

**Figure 4:**
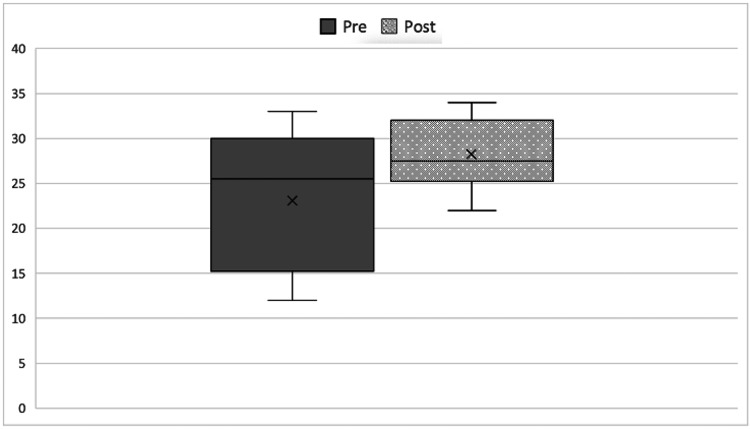
Surgical skills according to mean Objective Structured Assessment of Technical Skill (OSATS) score pre- and post-training.

The mean score according to the OSATS scale for surgical skills increased from 23.10 to 28.75, with the difference being statistically significant (p<0.001) (Figure 4).

**Figure 5:**
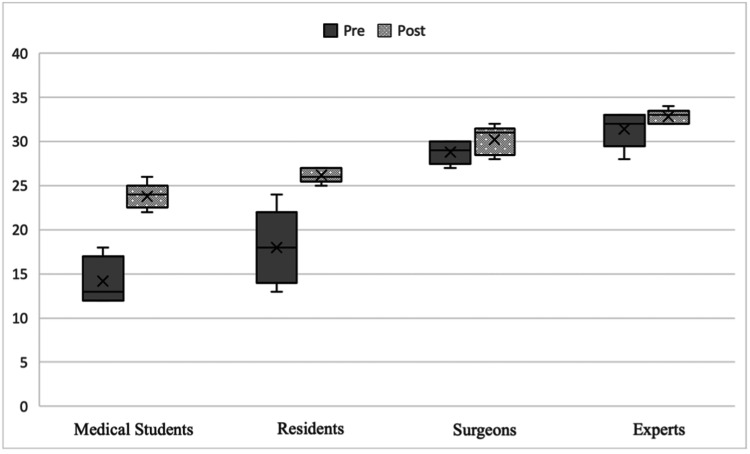
Mean Objective Structured Assessment of Technical Skill (OSATS) score pre- and post-training according to experience.

When comparing skills according to experience, a statistically significant difference was observed for both students (p=0.04) and residents (p=0.04), but not for surgeons (p=0.11) and experts (p=0.13) (Figure 5).

**Figure 6:**
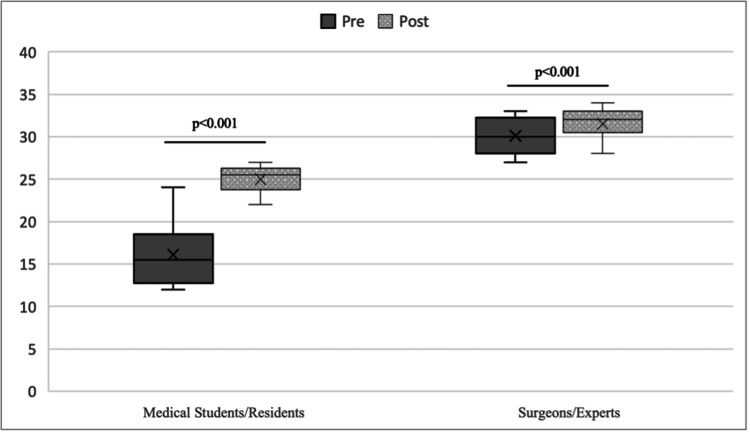
Mean Objective Structured Assessment of Technical Skill (OSATS) score pre- and post-training regrouped according to experience.

The mean skill score considering the categories students/residents and surgeons/experts, the comparison between pre- and post-training was significant (p<0.001). Within groups, we also found significant results, being even higher among students and residents (p<0.001) (Figure 6).

When comparing hermeticity, no statistically significant difference was found for the total sample (p=0.33). Among the students, two achieved this objective in the pre-test and four in the post-test. Meanwhile, four of the residents were able to perform a tight seal in the pre- and all in the post-test.

Regarding permeability, a statistically significant difference was found for the total sample (p=0.02). When analyzed according to experience, in the pre-test, three students and four residents achieved it, while in the post-test all performed a permeable anastomosis.

**Figure 7:**
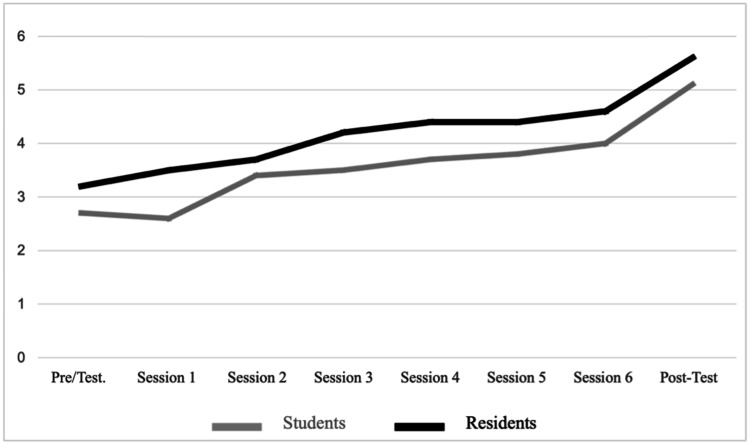
Perception of autonomy in students and residents pre- and post-training.

The perception of autonomy increased among students and residents without showing changes among surgeons and experts. Although both students and residents had increased confidence at the end of the program, the assignment of higher scores predominated among the latter (Figure 7).

## Discussion

The decrease in the number of open surgeries associated with the increase in laparoscopic surgery and other minimally invasive techniques, combined with changes in residency regulations, has negatively impacted residents' perception of autonomy at the end of their training [8,15-18]. Surveys reveal that 40% of them do not trust their surgical skills after five years of training [17]. In this context, simulation takes on a leading role, although there is limited data on its effectiveness and methodology in open surgery [19,20]. Indeed, the value and validity of laparoscopic simulation are reflected in the fact that the American Board of Surgery implements it in its curriculum called Fundamentals of Laparoscopic Surgery [21], and the American College of Surgeons required its implementation in the curriculum of all residency programs a year later, starting in 2009 [20]. In our setting, the Argentine Surgery Association has considered the implementation of surgical simulation essential for accrediting general surgery residencies since 2015 [22].

The proposed program successfully conveys the necessary concepts for crafting a quality intestinal anastomosis in a scenario that adds the difficulty of the environment, similar to what occurs in vivo. These concepts were selected for being the most complex to acquire, both technically and cognitively. It is noteworthy that participants with no experience and without a new interaction with the intestine after the pre-test were able to successfully perform their anastomosis. At the other end, experts also improved their performance, proving to be an effective review that allows improvement without the need to use biological tissues.

The methodological design followed classic guidelines with the presence of a pre-test, a post-test, and the participation of experts [23,24]. Unlike other studies, we included medical students, who not only contribute to their own education but also provide us with a complete range of results, from no experience to maximum experience [19,24-27]. Our training significantly improved the results of permeability, so we consider it essential to convey through scenarios, especially concepts such as tissue handling, suture depth, and three-dimensionality. Although the results of tightness were not significant, all participants achieved the objective. As a limitation for analysis, it should be mentioned that we did not measure the pressure to which we subjected each anastomosis to burst.

The program shows significant overall effectiveness in improving operative times, surgical skills, and hermeticity of anastomoses, similar to other experiences in open simulation, although not comparable due to methodological differences. Egle et al., in a similar study, showed that the OSATS score increased from 14.9 to 15.6, and the anastomosis time decreased from 23 to 18 minutes. It is noteworthy, compared to our work, that the OSATS score is globally lower, while the operative time is shorter. It should be noted that in said article, the anastomosis was end-to-end and single-layer, only residents participated, and the evaluation was not blinded [19]. On the other hand, Mackenna et al. recently demonstrated that training improved the confidence of the participating residents, although they did not evaluate other variables such as skills, time, or functionality of the anastomosis [25]. The use of widely available materials and standard surgical instruments makes the proposed model feasible in most surgical training centers. However, a formal cost analysis was not conducted, and variability in local resource availability may affect its replicability [28]. 

Regarding evaluation, we used the OSATS scale, considered the gold standard for measuring surgical skills [5,12-14,29]. Additionally, this work adds participant anonymity and blinded evaluation through videos, enhancing the validity of our results compared to previous experiences [16,19,26]. On the other hand, the Likert scale, which is often used as a tool for continuous improvement of simulation programs, allowed us to measure the participants' perception of autonomy [25]. This variable showed an increase in confidence levels among students and residents, suggesting that the proposed training could reduce the number of procedures needed to complete the learning curve. The validation of this tool as a measure of autonomy remains pending.

## Conclusions

The training program in open simulation of intestinal anastomoses demonstrated improvements in surgical skills, operative time, and perceived autonomy across all groups, with the greatest positive impact observed in students and residents. Despite the small sample size, these findings highlight the value of reinforcing open surgical competencies through structured simulation programs, particularly in the context of the minimally invasive era.

## Appendices

Appendix 1

**Table 2:** Likert Scale Perception of Autonomy by Participants

How prepared do you feel to perform an open intestinal anastomosis?						
0	1	2	3	4	5	6
Not prepared	Could not do it even with help	Could do it with prior practice	Could do it with a lot of help	Could do it with little help	Could do it with supervision	Feel fully prepared
